# Changes in Bird Functional Diversity across Multiple Land Uses: Interpretations of Functional Redundancy Depend on Functional Group Identity

**DOI:** 10.1371/journal.pone.0063671

**Published:** 2013-05-17

**Authors:** Gary W. Luck, Andrew Carter, Lisa Smallbone

**Affiliations:** Institute for Land, Water and Society, Charles Sturt University, Albury, New South Wales, Australia; University of Kent, United Kingdom

## Abstract

Examinations of the impact of land-use change on functional diversity link changes in ecological community structure driven by land modification with the consequences for ecosystem function. Yet, most studies have been small-scale, experimental analyses and primarily focussed on plants. There is a lack of research on fauna communities and at large-scales across multiple land uses. We assessed changes in the functional diversity of bird communities across 24 land uses aligned along an intensification gradient. We tested the hypothesis that functional diversity is higher in less intensively used landscapes, documented changes in diversity using four diversity metrics, and examined how functional diversity varied with species richness to identify levels of functional redundancy. Functional diversity, measured using a dendogram-based metric, increased from high to low intensity land uses, but observed values did not differ significantly from randomly-generated expected values. Values for functional evenness and functional divergence did not vary consistently with land-use intensification, although higher than expected values were mostly recorded in high intensity land uses. A total of 16 land uses had lower than expected values for functional dispersion and these were mostly low intensity native vegetation sites. Relations between functional diversity and bird species richness yielded strikingly different patterns for the entire bird community vs. particular functional groups. For all birds and insectivores, functional evenness, divergence and dispersion showed a linear decline with increasing species richness suggesting substantial functional redundancy across communities. However, for nectarivores, frugivores and carnivores, there was a significant hump-shaped or non-significant positive linear relationship between these functional measures and species richness indicating less redundancy. Hump-shaped relationships signify that the most functionally diverse communities occur at intermediate levels of species richness. Interpretations of redundancy thus vary for different functional groups and related ecosystem functions (e.g. pollination), and can be substantially different to relationships involving entire ecological communities.

## Introduction

Land-use change is one of the major drivers of biodiversity loss globally [1], [2]. While the impacts of land-use change on species richness (SR) and diversity have attracted substantial attention, greater emphasis is now being placed on the implications of land-use change for functional diversity (FD); an approach that links alteration in ecological community structure (e.g. species identity and population abundance) with the consequences for ecosystem function [3]. FD represents the functional differences among species [4] and captures the range, distribution and abundance of trait values of species in a community [5]–[7]. A functional trait is any morphological, physiological, phenological or behavioural (in the case of animals) characteristic of an individual [8], [9].

Research on FD includes observational studies of changes in diversity across environmental gradients or with land-use change [10]–[12], and small-scale manipulative experiments that test the impact of changes in species and FD on ecosystem functions such as biomass production [13]. These latter studies generally find that declines in FD result in disruptions to particular ecosystem functions. This relationship is likely to exist also at larger scales with major implications for humanity because certain functions are crucial to maintain productivity and ensure the flow of benefits (through ecosystem services) to humans (e.g. food production). Meta-analyses of changes in FD with land-use intensification generally show that increased intensification reduces FD, although this relationship can vary depending on land-use type, taxonomic group and FD measure [12], [14].

One of the major influences on interpreting FD relationships is the metric used to measure FD [15]–[17]. Mouchet et al. [18] argued that there are three major dimensions to functionality that need to be considered – functional richness, functional evenness and functional divergence (described below in ‘Functional Diversity Indices’). Pakeman [19], in one of the few studies comparing the performance of multiple indices in the context of land-use intensification, found that the same plant communities occupying the same environmental gradient yielded different patterns in functional richness, evenness and divergence. Therefore, measuring different components of functionality should result in a greater understanding of the impacts of land-use change on community assembly and ultimately ecosystem function.

A key issue of importance is the relationship between FD and SR [20], [21]. By examining this relationship, researchers can identify the presence of high or low functional redundancy in a community [5], [22]. High functional redundancy occurs when SR is high but FD is low owing to overlap in species traits. Here, the loss of some species may not result in the loss of FD or disrupt ecosystem functioning (assuming species loss is random). Functional redundancy is low when many species in a community are functionally ‘unique’ (i.e. no overlap in trait values) and here, species loss has major implications for ecosystem function. Yet, little is known about how redundancy varies across environmental gradients for the different dimensions of functionality, or when the emphasis is placed on particular functional groups that provide key ecosystem functions (e.g. frugivores that disperse seeds).

Functional traits may be split into response and effect traits. Response traits reflect the response of organisms to environmental change [23], [24]. A higher diversity of response traits in a community should, in theory, provide greater insurance (i.e. resilience; [25], [26]) against environmental perturbations causing complete community collapse because of an enhanced capacity for the community to adapt to various types of perturbations. Effect traits determine the effect an organism has on ecosystem functioning [23]. A community with higher levels of effect diversity should support a greater range of ecosystem functions or greater differential supply of particular functions. While either response or effect diversity could be interpreted in the context of resilience or redundancy, it is the interrelationships between response and effect traits that determine the impact of environmental change on ecosystem function [9], [27].

While much progress has been made in understanding the relationships among SR, FD and ecosystem function in small-scale, experimental studies, large-scale analyses across multiple land-uses in the same region using various FD metrics are lacking (see [19]). Moreover, studies of changes in FD with land-use intensification have sometimes, by necessity, included only a limited number of land-use types (e.g. Flynn et al. [12] compared changes in FD across ‘natural’, ‘semi-natural’ and ‘agricultural’ land uses). Also, research on FD and land-use change is dominated by studies of plants limiting the capacity to generalise about the impacts of change on a variety of ecosystem functions [28]. Here, we assess changes in the FD of bird communities across 24 different land uses to test the hypotheses that FD is higher in less intensively used landscapes and in landscapes with higher productivity in a particular land-use class (e.g. native vegetation). We document changes in response and effect diversity using four different FD metrics, and identify levels of functional redundancy for the entire bird community and for particular functional groups.

## Materials and Methods

### Ethics Statement

All field work was approved by the Charles Sturt University Animal Ethics Committee under permit numbers 04/031, 06/068, 09/062, 09/123 and 10/087. Permission to access conservation reserves was granted by the Victorian Department of Sustainability and Environment under permit numbers 10003799, 10004961, 10005535 and 10005663. Permission to access private property was granted by relevant land owners.

### Land Uses and Survey Data

**Figure 1:**
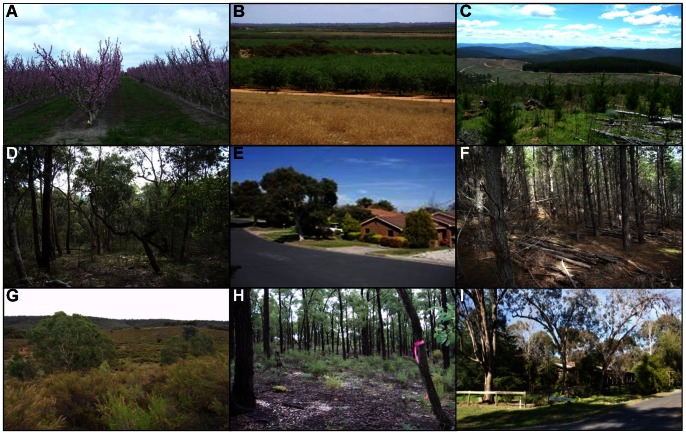
A selection of the 24 different land uses represented in this study. Apple orchard (A), almond orchard (B), young pine plantation (C), shrubby dry forest (D), medium density urban neighbourhood (E), mature, thinned pine plantation (F), shrub-dominated regrowth (G), tree-dominated regrowth (H), and low density urban neighbourhood (I).

The data used in our study were compiled from 10 years of bird surveys across 24 different vegetation and land-use types located in northern Victoria, Australia. Land uses ranged from native vegetation communities in large protected areas, native hardwood (e.g. *Eucalyptus camaldulensis*) and exotic softwood (*Pinus radiata*) plantations, urban neighbourhoods, regrowth on abandoned agricultural land, and various horticultural crops on private land (e.g. apples, almonds and vineyards; Figure 1; Table S1).

Vegetation types and land uses were aligned along a productivity/intensification gradient based on large-scale factors such as rainfall and similarity to existing native vegetation, and smaller-scale factors such as soil type and topographic position. In Australia, rainfall is a strong indicator of and very tightly linked to primary productivity [29], and soil type and topographic position are good indicators of soil fertility (e.g. run-on areas are generally more fertile than ridges in a given location). Native vegetation was always considered a less intensive land use than human-modified landscapes. Native vegetation types in higher rainfall areas were considered more productive than those in lower rainfall areas, while different vegetation types in the same rainfall band were ranked by small-scale productivity differences (e.g. higher rankings for run-on areas such as valleys compared to ridges with shallow soils). Modified land-uses were ranked based on rainfall, similarity to existing native vegetation, and other considerations of land-use intensity (see Table S1). These rankings were problematic in some cases; for example, comparing native hardwood plantations to native regrowth, or urban land uses to intensive horticulture. Therefore, our fine-scale rankings should be considered initial hypotheses of land-use impacts on FD that serve as useful points of departure when discussing changes in FD across land-uses.

We focussed on birds because they contribute to many ecosystem functions and fill a diverse range of ecological niches [30]. To improve the compatibility of data across land uses we applied the following restrictions to data inclusion: 1) including only bird abundance data collected using transect or point counts for a duration of 20 minutes (except for the three urban land-use categories, where surveys were completed in 12 minutes); 2) bird survey area covered between 1–2 ha ( =  a ‘plot’); 3) a total of six replicate plots included for every land use; and 4) each plot visited four times across two to four seasons (i.e. each land use was sampled 24 times). Further details of surveying in each land use can be found in related publications [9], [31], [32].

### Functional Diversity Indices

Mouchet et al. [18] reviewed the use of various FD metrics, arguing that most measures corresponded to one of three components of FD: functional richness, functional evenness (FEve) or functional divergence (FDiv). Laliberté and Legendre [17] introduced a further component – functional dispersion (FDis) – which is a weighted version of functional richness and similar to Rao’s quadratic entropy [33], the latter being described as representing a mix between functional richness and FDiv [18]. We quantified these four components of FD for our data set.

To represent functional richness, we used the dendogram-based FD measure of Petchey and Gaston [15] abbreviated as FDw. While this measure may not be the best choice in certain circumstances (e.g. when communities contain <10 species; [18]), it is one of the most commonly used measures of FD and facilitates comparison of our results with those of past studies. We weighted trait values by species abundance (see [34]) because, we argue, accounting for abundance is important when assessing the capacity of ecological communities to respond to environmental change or their impact on ecosystem functioning. This modification has a minor impact on the relevance of our results to those studies not incorporating species abundance because abundance-weighted and unweighted measures of FDw were highly positively correlated in our data set (*r* typically >0.95).

We used the measures of Villéger et al. [16] to represent FEve and FDiv, and Laliberté and Legendre [17] to represent FDis. Villéger et al. [35] introduced an additional measure – functional specialization – but we found that values of this measure were strongly positively correlated with those of FDis in our data set (*r* typically >0.8). FEve measures the evenness in the distribution of abundances across species in functional trait space [16], [36]. FEve is constrained between 0 and 1, whereby low values represent greater concentration of species along the functional axis and a higher proportion of abundances concentrated in a small compartment of functional trait space, and high values represent greater similarity in abundances and the distances among nearest neighbour species [16]. FDiv, also constrained between 0 and 1, measures ‘…how abundance is distributed within the volume of functional trait space occupied by species’ ([16], p. 2293). Laliberté and Legendre [17] argued that FDiv measures the distribution of species within the convex hull independent of its volume rather than the dispersion of species in trait space. Finally, FDis is a multivariate measure of the dispersion of species in trait space and represents ‘…the mean distance of individual species to the centroid of all species in the community’ ([17], p. 301).

In line with past research, we found that FDw was strongly positively correlated with SR, while FEve, FDiv and FDis were not (see also [17], [18]). Moreover, we found that all four measures were largely independent of each other (*r* typically <0.4), confirming that they represent different facets of FD [18]. So that the results from each index for a particular analysis were compared under the same circumstances, indices were calculated using the same set of traits and we ensured that there were never more traits than species included in the analysis for any given sample unit.

### Selection of Traits

The aim of our analysis was to examine the impact of land-use change and intensification on the FD of bird communities, and explore the implications of this change for ecosystem functioning. Functional traits were defined as either response or effect traits (see Introduction). Luck et al. [9] described the various functional traits for which data were collected, identified response and effect traits, and listed the sources of trait information relevant to the traits used in the current study. As is common in large-scale studies of animal communities, trait values were sourced from the literature rather than being measured directly in the field (which is extremely difficult when dealing with highly mobile animals occupying multiple landscapes). Also, animal-based studies of functional diversity generally consider behavioural characteristics (e.g. habitat use and foraging) of organisms as ‘traits’ [9]. Characteristics such as foraging behaviour or diet are crucial to understanding how an animal may respond to environmental changes and how it impacts on ecosystem function. This is fundamentally different to plant-based trait studies, which do not need to consider behavioural aspects of the focal organisms.

In the analysis of response traits, we aimed to determine how land-use change impacted on overall response trait diversity. That is, we did not focus on a particular environmental change that species may respond to, but rather wished to characterise their capacity to respond to land-use changes more generally. Higher levels of response trait diversity may help to buffer communities from various environmental changes in that more diverse communities should contain at least some species that are able to adapt to a particular environmental change. Similarly, we aimed to determine how land-use change affected overall effect trait diversity, which represented the capacity of bird communities to contribute to a range of ecosystem functions. In addition to these general characterisations, we focussed on the following four specific ecosystem functions that birds make important contributions towards: pollination; biological control (i.e. control of invertebrate pests); seed dispersal; and waste disposal (i.e. the disposal of animal waste, e.g. carcasses). A separate analysis was conducted for each function, and effect traits were chosen that were considered important in influencing the contribution of bird communities to these four functions.

Prior to final trait selection, we assessed correlations among traits and removed those with the least biological relevance to the current study [3]. We also excluded traits for which there were a large number of missing values (i.e. territoriality, mating system, vagility and tarsus length). From this reduced list, we selected the following response traits to characterise response trait diversity (see Table S2 for trait values for all species): body mass; clutch size; and habitat plasticity. Body mass is a key animal trait that is strongly related to various characteristics such as metabolic rate and life span [37]. It is also related strongly to the contribution of species to various ecosystem functions (e.g. the body size of insectivorous birds will impact on the amount and type of insects consumed thereby influencing biological control outcomes); therefore, it was included as a trait in all our analyses. Clutch size was used to represent the range of reproductive strategies and output (fecundity) occurring in a given bird community, as a diversity of strategies may be important for coping with environmental change [38]. Habitat plasticity is a continuous measure representing the level of habitat specialisation associated with a given species. Plasticity was derived by summing the frequency of occurrence of a species across 13 different habitat types and then adding the number of habitat types the species occurred in. Smaller values of this index represent species with more specialised habitat requirements and larger values represent habitat generalists (see [9]). A diversity of habitat use strategies should confer greater capacity to adapt to environmental change for a given bird community.

To characterise effect trait diversity, we selected the following traits: body mass; foraging behaviour plasticity; foraging location plasticity; foraging substrate plasticity; and diet plasticity. The impact of birds (and other fauna) on ecosystem function is related primarily to what they eat and how they procure their food [30] and traits related to resource acquisition can have a strong influence on biodiversity–ecosystem function relationships [12], [39]. Therefore, it was important to capture information on the foraging and dietary characteristics of bird communities, and a diversity of foraging and dietary strategies within a community represents the level of contribution made by species to overall ecosystem function. The various plasticity measures were calculated as for habitat plasticity; for example, foraging behaviour plasticity was calculated as the frequency with which a particular species used particular foraging behaviours plus the number of different foraging behaviours it used [9].

To assess FD related to a particular ecosystem function, we split bird communities based on diet. For pollination, we identified those species that include nectar in their diet (as these are most likely to contribute to pollination) and then calculated diversity measures for this dietary group only (nectarivores). The same approach was used for invertebrate pest control (species that include invertebrates in their diet – insectivores), seed dispersal (species that include fruit or seeds in their diet – frugivores/granivores) and waste disposal (species that include carrion or animal waste in their diet – carnivores/omnivores).

We calculated the diversity of effect traits for each dietary group that contributed to each ecosystem function, selecting those traits that were important to the function of interest. Given that many plots contained only a few species in each dietary group, we focussed on the most important traits. We selected four traits for invertebrate pest control, and two traits for the other three functions as follows: invertebrate pest control – body mass, foraging behaviour plasticity, foraging location plasticity and foraging substrate plasticity; pollination – body mass and foraging location plasticity; seed dispersal – body mass/wing span (related closely to body mass, but a better measure of movement capacity of a species, which is a critical factor for seed dispersal; [40]) and foraging location plasticity; and waste disposal – body mass and habitat plasticity (since foraging for waste occurs mostly on the ground, foraging location plasticity was not considered the most important measure). Plots with no species or only one species in each dietary group were given a FD value of ‘0’ for each diversity measure.

### Data Handling and Analysis

All FD indices were calculated using the software program FDiversity [34]. Prior to analyses, each trait measure was standardised to a mean of 0 and a standard deviation of 1. We used the unweighted pair-group method with arithmetic mean based on a Gower distance matrix to calculate FDw, as this consistently yielded the highest cophenetic correlation (typically >0.8) after testing among different distance matrices and linkage options.

To test if observed measures of FD were less than or greater than what would be expected by chance given the SR of each community, we used randomisations to generate a distribution of expected values for each FD measure [12], [19]. This is especially important for FD measures that are related closely to SR, such as FDw, whereby it is necessary to examine changes in FD independent of changes in SR [12]. We generated 999 random communities where SR in each plot remained constant (i.e. the same as observed values). Species were selected from the regional pool of species (those occurring across all land-uses) without replacement and randomly assigned to each plot. Abundances for each species were also chosen at random (without replacement) from the distribution of abundances occurring in each land use [41]. Each FD measure based on response or effect traits was then calculated for each random community to yield a distribution of 999 values for each measure. Observed values were considered significantly different to random values if they were ranked higher or lower than the 25^th^ or 975^th^ ranked random value, respectively.

SR was regressed against each FD index using linear or non-linear (quadratic) regression for the entire bird community (based on effect traits only; relationships were the same for analyses using response traits (unpublished data)) and for each dietary group. Quadratic relationships were accepted over linear relationships if they improved model fit (increased the *R* ^2^ value) and parameter estimates were significant (other non-linear relationships were explored, but quadratic regressions always yielded the highest *R* ^2^ value).

## Results

### Functional Diversity across Land Uses

#### Response and effect traits

**Figure 2:**
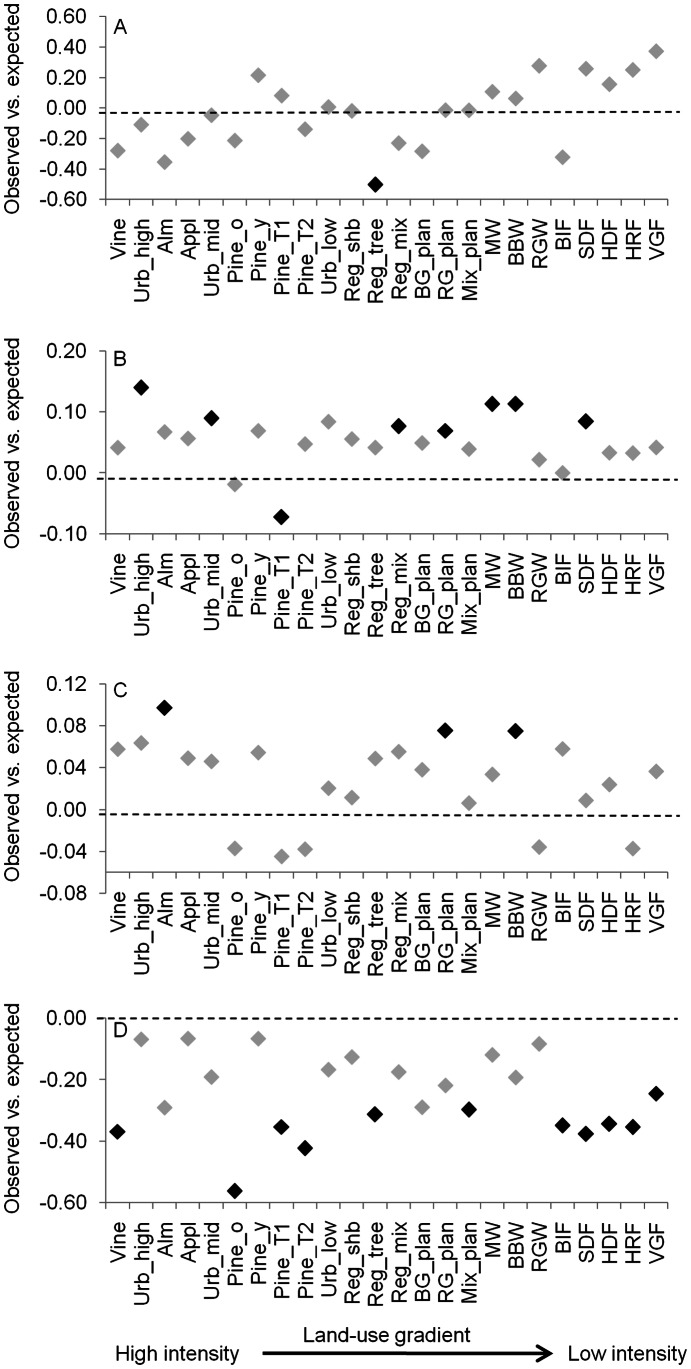
The difference between observed and expected values for each FD metric based on response traits. Shown is the magnitude of the difference for each land-use aligned along the land-use intensity gradient. A black diamond indicates a significant difference (α = 0.05). A = FDw; B = FEve; C = FDiv; D = FDis.

**Figure 3:**
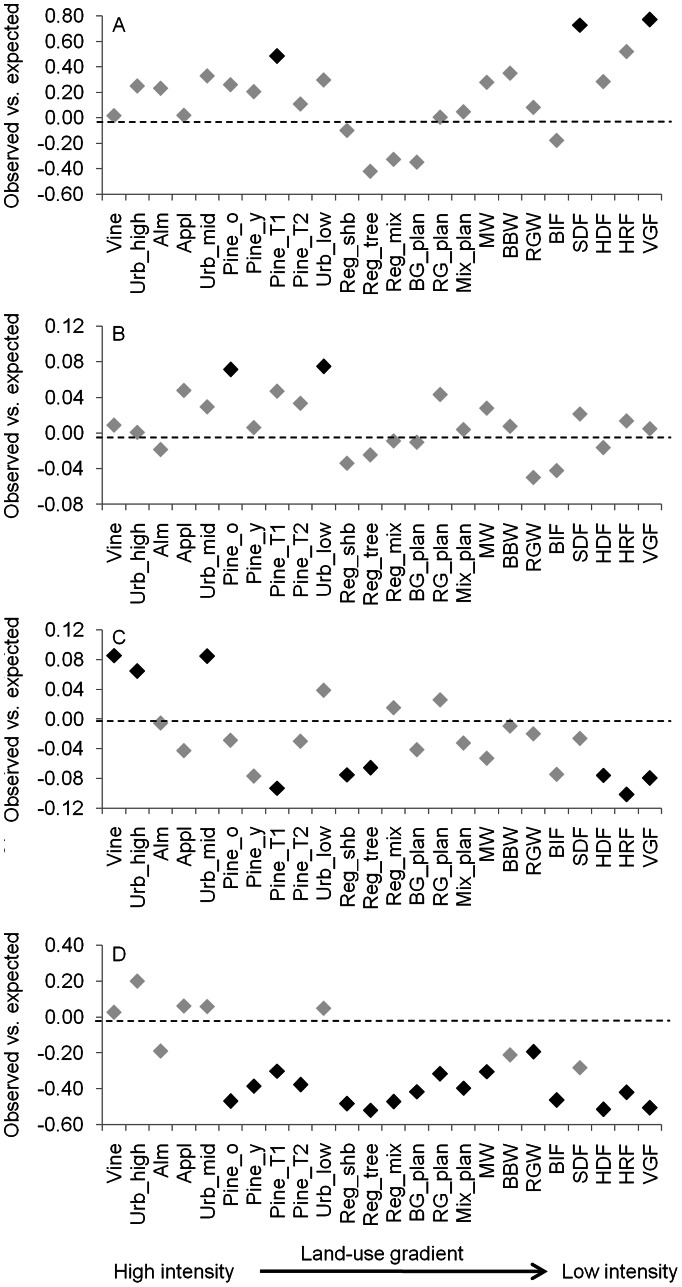
The difference between observed and expected values for each FD metric based on effect traits. Shown is the magnitude of the difference for each land-use aligned along the land-use intensity gradient. A black diamond indicates a significant difference (α = 0.05). A = FDw; B = FEve; C = FDiv; D = FDis.

Bird species richness generally increased from high to low intensity land uses, although some modified land uses (e.g. almond plantations) had relatively high species richness, while some native vegetation types (e.g. box-ironbark forest) had relatively low richness (Figure S1). Across the land-use gradient, FDw increased from heavily modified land-uses to native vegetation types for both response and effect traits (Figures S2 and S3). In most cases, observed values of FDw did not differ significantly from expected values. The exceptions were regrowth sites dominated by trees, which had significantly lower FDw than expected based on response traits, and mature, thinned pine plantations, and native shrubby dry forest and valley grassy forest, which had significantly higher FDw than expected based on effect traits (Figures 2a and 3a).

In contrast, FEve, FDiv and FDis based on either response or effect traits tended to decline slightly from modified land-uses to native vegetation (that is, an inverse relationship with intensification), or showed no clear trend (Figures S2 and S3). Nevertheless, there were clear departures from expectation among these FD indices. For example, seven land uses had higher than expected values of FEve based on response traits, five of which were native vegetation types (Figure 2b). For FDiv, six land uses (including three native vegetation types) had lower than expected values based on effect traits (Figure 3c). However, the most striking trend in these comparisons was that 46% and 67% of land-uses had lower than expected values for FDis based on response or effect traits, respectively (Figures 2d and 3d). This was particularly true among less intensive land uses (e.g. native vegetation).

Values of FDw based on response or effect traits were strongly related (*r* = 0.99), likely owing to the strong influence of SR on this measure (see below). However, values for the other indices were not strongly related, suggesting a focus on response or effect traits will capture different areas of functional space (FEve, *r* = 0.17; FDiv, *r* = 0.43; FDis, *r* = 0.33).

#### Diet groups

Assigning birds to dietary groups aligned with important ecosystem functions yielded variable patterns in FD across the land-use gradient. Patterns for insectivores largely mirrored those for the entire bird community based on response and effect traits, likely because many of the species in our communities consumed invertebrates (Figure S4). For the other three dietary groups, there was not a clear trend of increasing FDw with decreasing land-use intensification, though the highest values of FDw were generally recorded in native vegetation types (Figures S5, S6 and S7). In contrast to the results for all species and insectivores, values of FEve in native vegetation tended to be higher than or equivalent to values in other land uses for nectarivores, frugivores/granivores and carnivores/omnivores. This was true also for values of FDiv. Values for FDis were, however, quite variable across the land-use gradient.

### Relations with Species Richness

**Figure 4:**
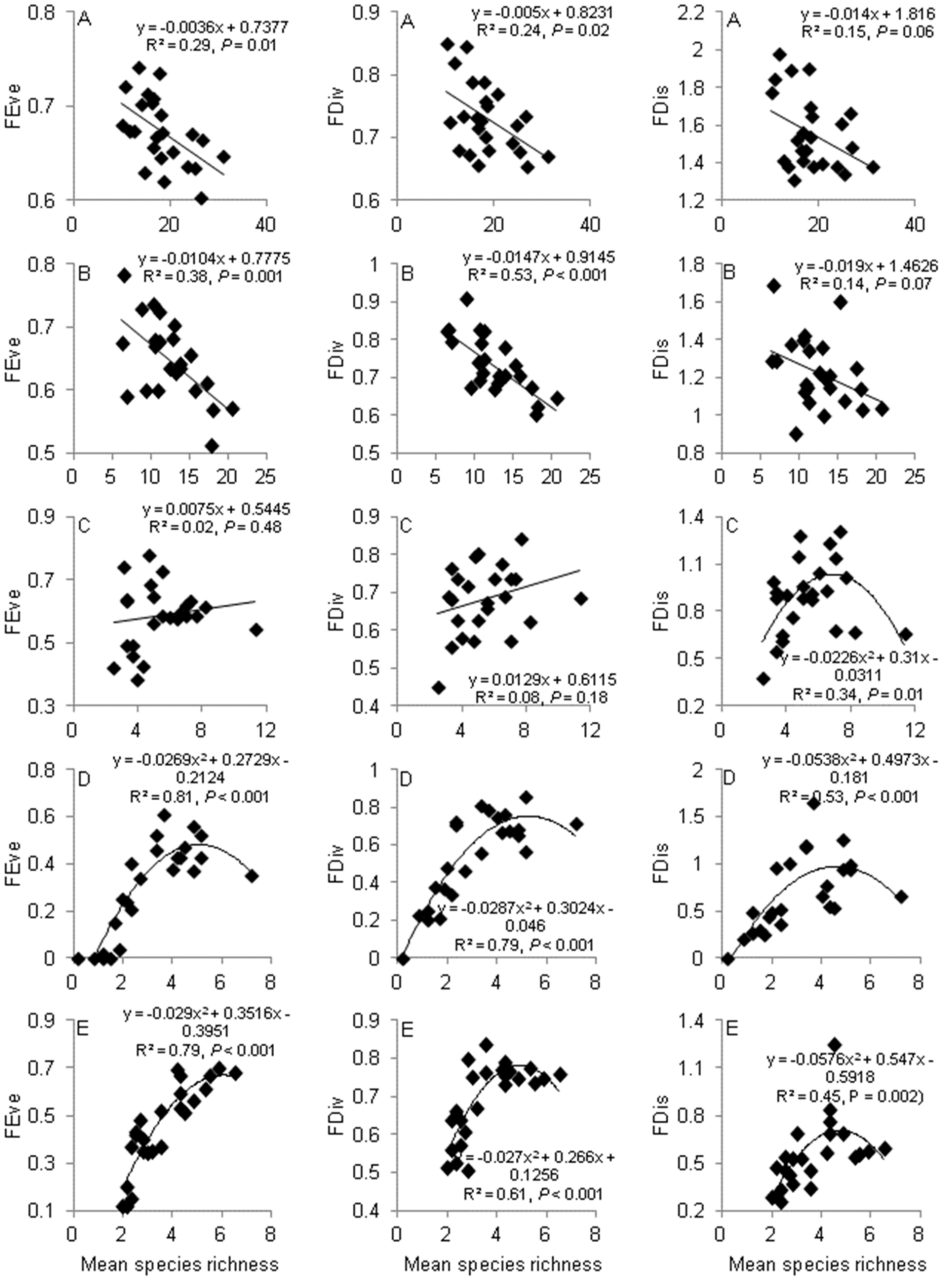
Relations between SR and FD in each land use. Shown are the results for FEve, FDiv and FDis for the entire bird community (A), insectivores (B), frugivores/granivores (C), nectarivores (D) and carnivores/omnivores (E).

Relations between the indices of FD and SR yielded strikingly different patterns for the entire bird community vs. different dietary groups with major implications for interpretations of functional redundancy. Values for FDw were always strongly positively related to SR, likely owing to the strong influence of SR on the calculation of FDw (Figure S8). For the entire bird community, FEve, FDiv and FDis showed a linear decline with increasing SR suggesting substantial functional redundancy across bird communities (Figure 4a). This was true also for insectivores, the dietary group with the largest number of species (Figure 4b). However, and most strikingly, relations between FEve, FDiv and FDis and SR for the remaining three dietary groups yielded significant quadratic (mostly hump-shaped) or non-significant positive linear relationships (Figures 4c, 4d and 4e). That is, in many cases, with increasing SR, FD increased – to a point – then plateaued or declined.

The differences between the entire bird community and the dietary groups are not an artefact of trait selection, because the relationships hold when recalculating the FD indices for the entire bird community using only the traits used for the dietary groups. However, these relationships are influenced by the lower SR in the dietary groups. Random draws from the entire species pool (i.e. across all diet groups) constrained to the same number of species occurring in a particular diet group, yields flat or positive linear relationships between SR and FEve, FDiv and FDis, although we did not record any hump-shaped relationships for these randomly generated communities (unpublished data).

## Discussion

### Functional Diversity across Land Uses

FDw increased with decreasing land-use intensity, but in most cases observed values did not differ significantly from expected values given the species richness in each community. Our results are broadly consistent with the meta-analysis of Flynn et al. [12] who found that changes in species richness and FD (measured using the Petchey and Gaston [15] index) were largely congruent, although their results also showed that in over one quarter of bird and animal communities, FD was lower than expected by chance in higher intensity agricultural land uses. Only one of our land uses (regrowth dominated by native trees) yielded lower than expected FDw considering both response and effect traits, while three land uses (mature thinned pine plantations and two native forest types) had higher than expected values. The congruence between observed and expected FDw values across most land uses suggests that changes in species richness dominate changes in FD for this particular index.

Various patterns in FD with changing land-use intensity were observed across the other three indices. Response diversity based on FEve was higher than expected by chance in 29% of land uses, including five native vegetation types. This suggests that bird communities in at least some native vegetation types may be more resilient to environmental change. That is, these locations are characterised by bird communities with greater response diversity and, across species, there is greater capacity to adapt to a broader range of environmental perturbations. However, this interpretation is not supported by the results for FDis, where native vegetation types often had lower than expected values. These results broadly agree with the meta-analysis of Laliberté et al. [14] who found that response diversity (measured using FDis) was commonly lower than expected in the least modified land uses. For effect diversity, no low intensity land uses had higher than expected values for any index of FD expect FDw, while five modified land uses (including urban areas, vineyards and pine plantations) had higher than expected values for FEve and FDiv. As for response diversity, effect diversity based on FDis was lower than expected for many land uses, particularly low intensity ones. The general conclusion from these analyses is that the FD of bird communities across many different land uses is mostly consistent with random expectations generated from simulated communities with the same species richness, except for FDis values which are mostly lower than expected especially for native vegetation types.

Changes in the FD of the four dietary groups across land uses were variable. Insectivores followed the patterns for all species with indications of a high level of redundancy among this dietary group (see below) and the capacity to adapt to various landscape modifications (see also [12]). For nectarivores, species richness and FD were generally lower in modified land uses compared to native vegetation types. This suggests that pollination by birds may be disrupted in these land uses. Our results are contrary to Flynn et al. [12] who found that pollinating birds often persisted in agricultural landscapes (based on a meta-analysis of studies from Central America to the northern United States). Among the modified land uses, urban areas maintained relatively high FD regardless of the index used to calculate this measure. In our study area, urban landscapes may be able to support higher numbers of nectarivores, and thus maintain pollination, owing to substantial plantings of nectar-bearing native trees and shrubs, and retention of native remnant vegetation close to human settlements [32]. There was little indication that seed dispersal would be disrupted by land-use modification, as the FD of frugivores/graniviores was, on average, similar between native vegetation and modified land uses. However, averaged across all modified land uses, the values of FDw, FEve and FDiv were generally lower for carnivores/omnivores compared to the values from native vegetation, suggesting the ecosystem function of waste disposal is at risk with increasing landscape modification.

Defining traits as either response or effect traits facilitated the identification of land-uses with high or low response or effect diversity, respectively. For example, redgum plantations and low density urban areas had high FEve values for both response and effect traits suggesting greater evenness in abundances among species and more regular spacing in functional trait space compared to other land uses (Table S3). In comparison, mature thinned and old unthinned pine plantations had high FEve values for effect traits, but low values for response traits suggesting unevenness in species abundances and irregularity in spacing when considering the bird community’s capacity to respond to environmental change. The interrelationships between response and effect traits are crucial to understanding how environmental change may impact on ecosystem function [9], [27]. For example, a given environmental perturbation may result in greater species loss from a community with low response diversity, but if effect diversity is high this perturbation may have little impact on ecosystem functioning overall. Conversely, if response diversity is high, a community may adapt readily to environmental changes, but the loss of even a few species may have major implications for ecosystem function if effect diversity is low. The most robust communities would be those with high levels of both response and effect diversity.

### Relations with Species Richness

Species richness had a positive linear or saturating relationship with FDw and we suggest measures of FDw should not be used to interpret the likelihood of functional redundancy in a community given its strong association with species richness. Indeed, the three other FD indices yielded completely different interpretations of redundancy. For the entire bird community, FEve and FDiv had significant negative relationships with species richness. This means, in the context of FEve, new species added to a community create greater unevenness in the distribution of abundances across species. This suggests that the new species are functionally similar to those already occurring in the community. Similarly, for FDiv, the addition of new species leads to a more concentrated distribution of species within the convex hull.

Our results are contrary to the results of Pakeman [19] who reported no strong relationship between FEve and FDiv and plant species richness in primarily agricultural land uses in Scotland. However, it is consistent with the results of Gerisch et al. [42] who found a negative correlation between ground beetle species richness and FEve and FDiv in grasslands in Germany. Strong negative relationships between species richness and functional diversity indices, as found for entire bird communities in our study, suggest substantial functional redundancy in communities as species richness increases across land uses. That is, when landscape modification reduces species richness, functionally redundant species are lost first (assuming species loss is random). These relationships are consistent when considering the insectivore dietary group and suggest resilience in the maintenance of related ecosystem functions (e.g. invertebrate pest control) when species are lost from the community (see also [12]).

However, interpretations regarding functional redundancy differ from above when the focus is on nectarivores, frugivores/granivores and carnivores/omnivores. In these cases, relations between species richness and FEve, FDiv and FDis are either weakly positive (essentially no relationship), saturating or hump-shaped. A flat relationship between species richness and FD suggests less functional redundancy than a negative relationship (although more redundancy than a strongly positive one). That is, as species richness increases FD remains constant. A saturating or hump-shaped relationship suggests communities with low species richness contain functionally unique species, but as richness increases, more functionally redundant species are added to the community. In the latter situation, the most functionally diverse communities occur at intermediate levels of species richness.

From the perspective of ecosystem function, in communities with relatively low species richness, loss of additional species likely has substantial implications for the functions of pollination and waste disposal (and to a lesser degree, seed dispersal). Conversely, for insectivores, FD appears to be maintained even at relatively low levels of species richness suggesting relatively less threat to the disruption of, for example, the ecosystem function of invertebrate pest control when species are lost from bird communities owing to landscape modification. The positive relationships between species richness and the FD of nectarivores, frugivores/granivores and carnivores/omnivores should be interpreted with caution as they appear to be influenced by the low species richness occurring in each dietary group. Interestingly, Mason et al. [43] recorded positive and hump-shaped relationships, respectively, between the index of variance of FEve and FDiv and the species richness of lake fishes with total richness similar to what we recorded for the three dietary groups. Further work is required that examines the performance of different FD indices when species richness varies, especially within groups of species associated with particular ecosystem functions. This may be challenging in real ecological communities where the number of species that contribute to a given ecosystem function may always be limited.

### Conclusion

Large-scale studies such as ours, on mobile vertebrate communities, have important differences to small-scale, experimental studies (mostly conducted on plants). First, large-scale vertebrate studies are generally not able to conduct in-field tests of the relationships between species traits and ecosystem function in each landscape. For example, measuring just the trait of body mass for birds would require the capture of all species in a given community (and multiple individuals to get some estimate of variance) – an extremely difficult proposition given that some species are hard to catch and community composition changes seasonally meaning new species would need to be captured. Second, linking particular traits to particular functions requires detailed field studies that can span years of work even for a single species. For example, confirming bill morphology is important for pollination requires extensive work on diet, flower visitation rate, pollen transfer efficiency, seed set and germination success, and how this varies across bird species with different bill shapes.

Given these challenges, trait data for vertebrate studies is commonly sourced through extensive searches of primary (individual field studies) or secondary (summaries of multiple field studies) literature. Moreover, relationships between traits and ecosystem functions (or services) are inferred for a particular location based on decades of past research on vertebrate-ecosystem function dynamics. This is especially true when dealing with multiple communities and multiple functions. Although, some site-specific case-studies involving particular functions (e.g. biological control) are able to conduct in-field tests of the relationship between bird traits and ecosystem function. It is important to understand these differences when comparing the approach and results of studies like ours with more explicit in-field tests of trait–function relationships.

Irrespective of the above caveats, FD is increasingly recognised as a key predictor of ecosystem function [7], [44] and, by extension, if these functions benefit humans FD can also be considered an important predictor of ecosystem service provision (e.g. [45]). Our results indicate that landscape modification does not reduce the FD of bird communities in any consistent fashion, although certain land-use types have lower FD than is expected by chance. Moreover, FD was not reduced consistently for any particular dietary group suggesting that ecosystem services such as pollination or biological control may be retained even in highly modified landscapes. Yet, landscapes with low species richness appear to have low functional redundancy for bird groups that contribute to the key services of pollination, seed dispersal and waste disposal. Promoting greater richness within these groups would help to reduce the risk of complete ecosystem-service disruption in these landscapes.

## Supporting Information

Figure S1 **Bird species richness in each land use ordered from high intensity (Vine) to low intensity (VGF).** See Table S1 for land-use codes.
(TIF)

Figure S2 **Observed (diamonds) and random (squares) values for each FD metric based on response traits.** See Table S1 for land-use codes.
(TIF)

Figure S3 **Observed (diamonds) and random (squares) values for each FD metric based on effect traits.** See Table S1 for land-use codes.
(TIF)

Figure S4 **Observed values for each FD metric for insectivores based on effect traits.** See Table S1 for land-use codes.
(TIF)

Figure S5 **Observed values for each FD metric for nectarivores based on effect traits.** See Table S1 for land-use codes.
(TIF)

Figure S6 **Observed values for each FD metric for frugivores/granivores based on effect traits.** See Table S1 for land-use codes.
(TIF)

Figure S7 **Observed values for each FD metric for carnivores/omnivores based on effect traits.** See Table S1 for land-use codes.
(TIF)

Figure S8 **Relations between SR and FDw in each land use.** Shown are the results for the entire bird community (A), insectivores (B), frugivores/granivores (C), nectarivores (D) and carnivores/omnivores (E).
(TIF)

Table S1 **A description of the 24 different vegetation or land-use types in which birds were surveyed.**
(DOC)

Table S2 **Trait values for all bird species included in this study.**
(DOC)

Table S3 **Land uses for which values of each FD metric were in either the highest or lowest quartile of values for response or effect traits.**
(DOC)
