# Supplementary material for: Changes in Bird Functional Diversity across Multiple Land Uses: Interpretations of Functional Redundancy Depend on Functional Group Identity
Source: PLoS One. 2013 May 17;8(5):e63671. doi: 10.1371/journal.pone.0063671 (PMC3656964; doi:10.1371/journal.pone.0063671)
Supplement: Table S1 — A description of the 24 different vegetation or land-use types in which birds were surveyed. (DOC) [file pone.0063671.s009.doc]

Table S1. A description of the 24 different vegetation and land-use types in which birds were surveyed (ranked from the lowest to highest intensity).

| Vegetation/land-use type (code) | Description | Productivity/intensification gradient rank |
| --- | --- | --- |
| Native vegetation types | | |
| Valley grassy forest (VGF) | Valley/footslope; common overstorey species *Acacia melanoxylon*, *A. dealbata*; common shrub species *A. rubida, Cassinia aculeata* | 1 – mean annual rainfall 1120 mm, run-on area, deep soils, often close to water source |
| Herb-rich foothill forest (HRF) | Lower slope/valley; common overstorey species *Eucalyptus radiata, A. melanoxylon, A. dealbata*; common shrub species *Cassinia longifolia, Coprosma quadrifida* | 2 – mean annual rainfall 1120 mm, run-on area, moderately deep soils |
| Heathy dry forest (HDF) | Moderate slope/ridge; common overstorey species *E. macrorhyncha, E. polyanthemos*; common shrub species *Brachyloma daphnoides, Hibbertia riparia* | 3 – mean annual rainfall 1120 mm, run-off area, skeletal sandy soils |
| Shrubby dry forest (SDF) | Upper slope/ridge; common overstorey species *E. dives, E. mannifera, E. radiata, A. melanoxylon*; common shrub species *A. rubida, Coprosma hirtella* | 4 – mean annual rainfall 1120 mm, run-off area, shallow, sandy soils |
| Box-ironbark forest (BIF) | Undulating flats, rocky areas; common overstorey species *E. Tricarpa*, *E. macrorhyncha, E. Polyanthemo;* common shrub species *A. pycnantha, Cassinia acuarta* | 5 – mean annual rainfall 500 mm, shallow, rocky soils |
| Red gum woodlands (RGW) | Lake and river margins; open woodland dominated by an overstorey of *E. camaldulensis* | 6 – mean annual rainfall 310 mm, run-on areas, deeper soils, near water sources |
| Black box open woodlands (BBW) | Lower slopes; open woodland with an overstorey of *E. largiflorens* and *Chenopod spp.* shrubs | 7 – mean annual rainfall 310 mm, lower slopes, clay soils, sometimes near water |
| Mallee woodlands (MW) | Upper slopes/ridges; common overstorey species *E. gracilis, E. incrassate*; common understorey species *Triodia scariosa* | 8 – mean annual rainfall 310 mm, run-off areas, upper slopes, sandy soils |
| Human land-use types | | |
| Hardwood plantation – mixed species (Mix_plan) | Mixed eucalypt planation with overstorey species of *E. polyanthemos, E. melliodora, E. cladocalyx, E. camaldulensis, E. microcarpa* | 9 – mean annual rainfall 600 mm, mix of endemic and non-endemic native eucalypt species, stocking density 650 stems ha^-1^ |
| Hardwood plantation – red gum (RG_plan) | Monoculture eucalypt plantation of *E. camaldulensis* | 10 – mean annual rainfall 600 mm, endemic species monoculture, stocking density 1000 stems ha^-1^ |
| Hardwood plantation – blue gum (BG_plan) | Monoculture eucalypt plantation of *E.* globulus | 11 – mean annual rainfall 600 mm, monoculture of native, but non-endemic species, stocking density 800 stems ha^-1^ |
| Regrowth sites – shrub and tree (Reg_mix) | Mixed regrowth of *E. microcarpa, Cassinia arcuata, A. pycnatha, A. paradoxa* | 12 – mean annual rainfall 500 mm, mix of endemic tree and shrub species |
| Regrowth sites – tree (Reg_tree) | Regrowth dominated by the overstorey species *E. microcarpa* | 13 – mean annual rainfall 500 mm , single endemic tree species |
| Regrowth sites – shrub (Reg_shb) | Regrowth dominated by the shrub species *Cassinia arcuata* | 14 – mean annual rainfall 500 mm , single endemic shrub species |
| Urban – low density (Urb_low) | Residential neighbourhoods in peri-urban locations on fringes of towns | 15 – mean annual rainfall 500–700 mm, low density housing (< 2 houses ha^-1^) interspersed with native and non-native plants |
| Softwood plantation – *Pinus radiata* – old thinned (Pine_T2) | Older plantation (25–28 years since planting), twice thinned | 16 – mean annual rainfall 1120 mm, pines interspersed with native plant species, stocking density after thinning 300–350 stems ha^-1^ |
| Softwood plantation – *Pinus radiata* – mature thinned (Pine_T1) | Mature plantation (19–20 years since planting), once thinned | 17 – mean annual rainfall 1120 mm, some understorey growth, stocking density after thinning 600–650 stems ha^-1^ |
| Softwood plantation – *Pinus radiata* – young unthinned (Pine_y) | Young trees (4–6 years since planting), unthinned | 18 – mean annual rainfall 1120 mm, some gaps in canopy with native shrub encroachment, stocking density 1000–1100 stems ha^-1^ |
| Softwood plantation – *Pinus radiata* – old unthinned (Pine_o) | Old trees (25 years since planting), never thinned | 19 – mean annual rainfall 1120 mm, little understorey growth, stocking density 900–1000 stems ha^-1^ |
| Urban – medium density (Urb-mid) | Residential neighbourhoods in suburban locations | 20 – mean annual rainfall 500–700 mm, medium density housing (4<6 houses ha^-1^), some native and non-native plants |
| Apple orchards (Appl) | Established orchards of various apple varieties | 21 – mean annual rainfall 500 mm, monoculture of apple trees with little other vegetation, stocking density 350 stems ha^-1^ |
| Almond orchards (Alm) | Established orchards of various almond varieties | 22 – mean annual rainfall 310 mm, shallow sandy soils, monoculture of almond trees with little other vegetation, stocking density 200 stems ha^-1^ |
| Urban – high density (Urb_high) | Residential neighbourhoods in urban (town centre) locations | 23 – mean annual rainfall 500–700 mm, high density housing (>8 houses ha^-1^), high impervious surface cover, mostly non-native vegetation |
| Vineyards (Vine) | Established vineyards of various grape varieties | 24 – mean annual rainfall 600 mm, monoculture of vines with little other vegetation, stocking density 700 stems ha^-1^ |
