# Supplementary material for: Changes in Bird Functional Diversity across Multiple Land Uses: Interpretations of Functional Redundancy Depend on Functional Group Identity
Source: PLoS One. 2013 May 17;8(5):e63671. doi: 10.1371/journal.pone.0063671 (PMC3656964; doi:10.1371/journal.pone.0063671)
Supplement: Table S2 — Trait values for all bird species included in this study. (DOC) [file pone.0063671.s010.doc]

Table S2. Trait values for all bird species included in this study.

| Scientific Name | Body mass (grams)^a^ | Clutch size^b^ | Habitat plasticity^c^ | Foraging behaviour plasticity^d^ | Foraging location plasticity^e^ | Foraging substrate plasticity^f^ | Diet plasticity^g^ |
| --- | --- | --- | --- | --- | --- | --- | --- |
| *Acanthagenys rufogularis* | 46.9 | 2.3 | 31 | 15 | 12 | 15 | 21 |
| *Acanthiza apicalis* | 7.4 | 2.6 | 35 | 9 | 13 | 15 | 10 |
| *Acanthiza chrysorrhoa* | 9.2 | 3.2 | 45 | 11 | 13 | 28 | 8 |
| *Acanthiza lineata* | 7.5 | 2.9 | 35 | 12 | 11 | 18 | 17 |
| *Acanthiza nana* | 6.4 | 3.1 | 39 | 11 | 14 | 12 | 8 |
| *Acanthiza pusilla* | 6.8 | 2.9 | 42 | 13 | 11 | 16 | 14 |
| *Acanthiza reguloides* | 7.8 | 3.6 | 26 | 11 | 13 | 22 | 11 |
| *Acanthiza uropygialis* | 6.4 | 3.3 | 27 | 12 | 15 | 17 | 8 |
| *Acanthorhynchus tenuirostris* | 11.8 | 2.2 | 42 | 9 | 14 | 13 | 8 |
| *Alisterus scapularis* | 233.1 | 4.5 | 29 | 15 | 15 | 24 | 20 |
| *Anas gracilis* | 494.4 | 8.8 | 20 | 20 | 10 | 20 | 10 |
| *Anas superciliosa* | 1060.2 | 8.6 | 27 | 21 | 9 | 18 | 14 |
| *Anthochaera carunculata* | 113.3 | 2.0 | 37 | 19 | 15 | 22 | 16 |
| *Anthochaera chrysoptera* | 70.9 | 1.7 | 37 | 14 | 15 | 28 | 15 |
| *Anthus novaeseelandiae* | 25.8 | 2.8 | 31 | 18 | 12 | 19 | 10 |
| *Aphelocephala leucopsis* | 13.3 | 3.6 | 30 | 8 | 13 | 18 | 13 |
| *Aquila audax* | 3462.4 | 1.7 | 49 | 31 | 11 | 14 | 10 |
| *Artamus cyanopterus* | 34.6 | 2.9 | 34 | 17 | 13 | 21 | 14 |
| *Artamus personatus* | 34.6 | 2.2 | 33 | 18 | 8 | 15 | 15 |
| *Artamus superciliosus* | 35.9 | 2.1 | 40 | 19 | 18 | 16 | 12 |
| *Aythya australis* | 872.8 | 11.5 | 16 | 18 | 10 | 15 | 15 |
| *Barnardius zonarius* | 143.8 | 4.3 | 32 | 12 | 13 | 27 | 21 |
| *Cacatua galerita* | 789.8 | 2.5 | 47 | 13 | 9 | 14 | 17 |
| *Cacatua roseicapilla* | 335.7 | 4.2 | 35 | 11 | 10 | 20 | 17 |
| *Cacatua sanguinea* | 382.4 | 2.6 | 27 | 9 | 9 | 22 | 13 |
| *Cacomantis flabelliformis* | 49.8 | 1.1 | 38 | 12 | 15 | 17 | 5 |
| *Callocephalon fimbriatum* | 256.3 | 2.0 | 22 | 5 | 13 | 13 | 13 |
| *Calyptorhynchus funereus* | 750.2 | 2.3 | 34 | 10 | 13 | 19 | 16 |
| *Carduelis carduelis* | 16.0 | 4.4 | 37 | 13 | 11 | 14 | 12 |
| *Carduelis chloris* | 26.1 | 4.6 | 29 | 12 | 13 | 14 | 17 |
| *Ceyx azurea* | 34.4 | 5.6 | 27 | 15 | 13 | 9 | 10 |
| *Chenonetta jubata* | 809.5 | 10.2 | 28 | 17 | 10 | 23 | 13 |
| *Cheramoeca leucosternum* | 14.1 | 4.6 | 36 | 5 | 5 | 5 | 5 |
| *Chrysococcyx basalis* | 18.9 | 1.0 | 45 | 5 | 19 | 17 | 11 |
| *Chrysococcyx lucidus* | 23.8 | 1.0 | 29 | 5 | 9 | 17 | 5 |
| *Chthonicola sagittata* | 13.3 | 2.9 | 18 | 11 | 13 | 25 | 13 |
| *Cincloramphus mathewsi* | 28.4 | 3.0 | 39 | 7 | 7 | 7 | 9 |
| *Climacteris erythrops* | 23.5 | 2.0 | 19 | 10 | 7 | 18 | 5 |
| *Climacteris picumnus* | 29.1 | 2.7 | 36 | 14 | 18 | 38 | 10 |
| *Colluricincla harmonica* | 66.3 | 2.9 | 45 | 15 | 12 | 23 | 18 |
| *Columba livia* | 350.4 | 1.8 | 20 | 10 | 9 | 14 | 12 |
| *Coracina novaehollandiae* | 117.1 | 2.5 | 45 | 18 | 14 | 20 | 14 |
| *Corcorax melanorhamphos* | 364.6 | 4.2 | 44 | 10 | 8 | 16 | 18 |
| *Corombates leucophaeus* | 22.4 | 3.3 | 28 | 13 | 12 | 26 | 10 |
| *Corvus coronoides* | 644.8 | 4.2 | 48 | 22 | 16 | 20 | 27 |
| *Corvus mellori* | 535.0 | 4.1 | 46 | 12 | 14 | 14 | 21 |
| *Cracticus nigrogularis* | 128.0 | 2.8 | 42 | 11 | 8 | 11 | 26 |
| *Cracticus torquatus* | 88.1 | 3.2 | 46 | 10 | 10 | 7 | 18 |
| *Cuculus pallidus* | 87.7 | 1.0 | 44 | 5 | 14 | 9 | 8 |
| *Dacelo novaeguineae* | 351.5 | 2.9 | 34 | 14 | 9 | 4 | 10 |
| *Daphoenositta chrysoptera* | 11.8 | 2.7 | 29 | 14 | 9 | 19 | 5 |
| *Dicaeum hirundinaceum* | 9.1 | 2.7 | 31 | 9 | 11 | 15 | 13 |
| *Egretta novaehollandiae* | 570.6 | 3.8 | 28 | 40 | 5 | 10 | 12 |
| *Elanus notatus* | 280.8 | 3.4 | 43 | 24 | 14 | 14 | 9 |
| *Entomyzon cyanotis* | 105.0 | 2.6 | 33 | 16 | 9 | 24 | 13 |
| *Eopsaltria australis* | 18.7 | 2.3 | 34 | 13 | 13 | 24 | 11 |
| *Ephthianura albifrons* | 13.7 | 3.0 | 38 | 14 | 10 | 14 | 8 |
| *Falco berigora* | 588.8 | 2.8 | 51 | 21 | 15 | 15 | 14 |
| *Falco cenchroides* | 170.2 | 3.8 | 37 | 36 | 13 | 14 | 10 |
| *Falcunculus frontatus* | 28.5 | 2.6 | 28 | 14 | 11 | 19 | 15 |
| *Fulica atra* | 547.1 | 5.7 | 5 | 20 | 10 | 10 | 14 |
| *Gallinula tenebrosa* | 525.1 | 7.8 | 28 | 22 | 10 | 20 | 30 |
| *Geopelia striata* | 49.2 | 2.0 | 36 | 5 | 5 | 15 | 8 |
| *Gerygone fusca* | 6.6 | 2.7 | 36 | 13 | 13 | 16 | 5 |
| *Gerygone olivacea* | 7.3 | 2.8 | 37 | 10 | 12 | 13 | 5 |
| *Glossopsitta concinna* | 76.1 | 2.0 | 34 | 5 | 7 | 14 | 17 |
| *Glossopsitta pusilla* | 39.4 | 4.0 | 31 | 5 | 5 | 15 | 10 |
| *Grallina cyanoleuca* | 87.3 | 3.8 | 50 | 14 | 9 | 9 | 11 |
| *Grantiella picta* | 21.6 | 3.6 | 25 | 11 | 8 | 17 | 11 |
| *Gymnorhina tibicen* | 278.7 | 3.4 | 50 | 20 | 13 | 14 | 26 |
| *Haliastur sphenurus* | 757.5 | 2.1 | 46 | 35 | 13 | 14 | 15 |
| *Hirundo neoxena* | 14.8 | 3.8 | 55 | 16 | 11 | 18 | 5 |
| *Hirundo nigricans* | 16.0 | 3.4 | 45 | 13 | 14 | 14 | 5 |
| *Hylacola pyrrhopygia* | 16.8 | 2.5 | 23 | 9 | 12 | 12 | 8 |
| *Lalage sueurii* | 25.3 | 2.4 | 39 | 15 | 15 | 21 | 18 |
| *Leucosarcia melanoleuca* | 429.0 | 2.0 | 20 | 9 | 8 | 16 | 11 |
| *Lichenostomus chrysops* | 17.3 | 2.4 | 36 | 10 | 12 | 24 | 20 |
| *Lichenostomus fuscus* | 25.7 | 2.7 | 29 | 15 | 12 | 21 | 11 |
| *Lichenostomus leucotis* | 24.7 | 2.1 | 34 | 11 | 12 | 24 | 15 |
| *Lichenostomus melanops* | 26.1 | 2.0 | 24 | 18 | 11 | 24 | 16 |
| *Lichenostomus ornatus* | 17.5 | 2.0 | 10 | 13 | 11 | 25 | 12 |
| *Lichenostomus penicillatus* | 19.2 | 2.3 | 36 | 17 | 14 | 23 | 16 |
| *Lichenostomus virescens* | 27.2 | 2.3 | 35 | 8 | 9 | 13 | 11 |
| *Malurus cyaneus* | 9.6 | 7.3 | 44 | 16 | 12 | 26 | 17 |
| *Malurus lamberti* | 8.0 | 2.9 | 31 | 10 | 13 | 25 | 8 |
| *Malurus splendens* | 9.0 | 2.9 | 27 | 9 | 13 | 21 | 8 |
| *Manorina melanocephala* | 61.2 | 2.8 | 34 | 14 | 12 | 23 | 18 |
| *Melanodryas cucullata* | 24.0 | 2.0 | 29 | 11 | 10 | 11 | 8 |
| *Melithreptus brevirostris* | 12.7 | 2.8 | 21 | 7 | 9 | 15 | 11 |
| *Melithreptus lunatus* | 14.5 | 2.7 | 29 | 11 | 13 | 17 | 10 |
| *Menura novaehollandiae* | 986.8 | 1.0 | 30 | 5 | 5 | 15 | 12 |
| *Merops ornatus* | 28.0 | 4.7 | 42 | 13 | 10 | 12 | 5 |
| *Microeca leucophaea* | 15.7 | 2.0 | 35 | 12 | 11 | 10 | 5 |
| *Myiagra cyanoleuca* | 17.5 | 3.0 | 32 | 11 | 13 | 15 | 8 |
| *Myiagra inquieta* | 20.9 | 3.3 | 26 | 15 | 8 | 18 | 5 |
| *Myiagra rubecula* | 12.6 | 1.9 | 29 | 13 | 13 | 12 | 7 |
| *Neochmia temporalis* | 10.7 | 5.2 | 48 | 12 | 10 | 17 | 13 |
| *Northiella haematogaster* | 89.0 | 5.0 | 22 | 3 | 11 | 16 | 21 |
| *Ocyphaps lophotes* | 204.4 | 2.0 | 32 | 5 | 5 | 5 | 13 |
| *Oreoica gutturalis* | 65.3 | 2.6 | 19 | 5 | 9 | 15 | 9 |
| *Oriolus sagittatus* | 95.5 | 2.2 | 30 | 13 | 8 | 19 | 20 |
| *Pachycephala inornata* | 31.0 | 3.1 | 17 | 5 | 10 | 12 | 11 |
| *Pachycephala pectoralis* | 26.2 | 2.2 | 43 | 19 | 12 | 19 | 10 |
| *Pachycephala rufiventris* | 24.9 | 2.6 | 42 | 15 | 11 | 22 | 14 |
| *Pardalotus punctatus* | 8.4 | 4.0 | 45 | 13 | 12 | 13 | 9 |
| *Pardalotus striatus* | 12.1 | 3.8 | 41 | 13 | 11 | 17 | 13 |
| *Passer domesticus* | 27.3 | 4.1 | 26 | 24 | 14 | 28 | 23 |
| *Petroica goodenovii* | 8.8 | 2.3 | 35 | 14 | 14 | 10 | 5 |
| *Petroica multicolor* | 12.9 | 2.8 | 30 | 15 | 16 | 20 | 5 |
| *Petroica phoenicea* | 13.3 | 3.0 | 38 | 11 | 13 | 19 | 5 |
| *Petroica rosea* | 8.2 | 3.0 | 22 | 16 | 15 | 12 | 5 |
| *Phalacrocorax melanoleucos* | 743.3 | 4.0 | 14 | 10 | 5 | 5 | 9 |
| *Phaps chalcoptera* | 332.1 | 1.8 | 35 | 5 | 8 | 8 | 14 |
| *Phaps elegans* | 211.5 | 2.0 | 31 | 5 | 5 | 15 | 5 |
| *Philemon citreogularis* | 64.5 | 2.4 | 26 | 12 | 11 | 20 | 16 |
| *Philemon corniculatus* | 103.6 | 3.0 | 35 | 10 | 6 | 16 | 22 |
| *Phylidonyris albifrons* | 17.4 | 2.0 | 27 | 11 | 12 | 9 | 11 |
| *Phylidonyris novaehollandiae* | 20.6 | 2.1 | 37 | 10 | 12 | 13 | 10 |
| *Platycercus elegans* | 133.2 | 4.4 | 35 | 7 | 14 | 19 | 21 |
| *Platycercus elegans* | 115.9 | 4.4 | 35 | 5 | 12 | 14 | 21 |
| *Platycercus eximius* | 104.7 | 5.6 | 33 | 10 | 11 | 24 | 21 |
| *Poliocephalus poliocephalus* | 240.5 | 3.4 | 18 | 14 | 5 | 10 | 5 |
| *Polytelis anthopeplus* | 177.9 | 4.7 | 26 | 9 | 9 | 14 | 19 |
| *Pomatostomus superciliosus* | 39.6 | 2.6 | 32 | 10 | 10 | 25 | 13 |
| *Psephotus haematonotus* | 62.8 | 5.0 | 36 | 5 | 10 | 10 | 14 |
| *Psephotus varius* | 61.5 | 5.5 | 23 | 5 | 13 | 15 | 16 |
| *Psophodes olivaceus* | 63.5 | 1.9 | 32 | 10 | 11 | 25 | 11 |
| *Ptilonorhynchus violaceus* | 218.8 | 1.8 | 30 | 11 | 13 | 21 | 26 |
| *Rhipidura fuliginosa* | 8.3 | 2.8 | 45 | 15 | 12 | 19 | 13 |
| *Rhipidura leucophrys* | 19.9 | 3.0 | 47 | 23 | 12 | 21 | 8 |
| *Sericornis frontalis* | 14.2 | 2.9 | 44 | 11 | 12 | 28 | 13 |
| *Smicrornis brevirostris* | 6.1 | 2.6 | 30 | 17 | 11 | 13 | 8 |
| *Stagonopleura guttata* | 17.6 | 4.7 | 34 | 11 | 9 | 14 | 11 |
| *Strepera graculina* | 331.6 | 3.1 | 31 | 22 | 18 | 44 | 24 |
| *Strepera versicolor* | 384.6 | 2.6 | 36 | 19 | 13 | 28 | 14 |
| *Streptopelia chinensis* | 160.5 | 1.9 | 15 | 5 | 5 | 5 | 9 |
| *Struthidea cinerea* | 131.8 | 4.1 | 32 | 21 | 14 | 8 | 21 |
| *Sturnus vulgaris* | 84.9 | 4.6 | 45 | 22 | 17 | 26 | 20 |
| *Taeniopygia guttata* | 12.2 | 4.5 | 36 | 11 | 5 | 11 | 13 |
| *Threskiornis spinicollis* | 1335.0 | 3.0 | 25 | 10 | 10 | 20 | 5 |
| *Todiramphus sanctus* | 43.3 | 4.3 | 28 | 21 | 20 | 22 | 9 |
| *Trichoglossus haematodus* | 131.4 | 2.0 | 44 | 9 | 7 | 16 | 19 |
| *Turdus merula* | 110.7 | 3.5 | 41 | 10 | 15 | 16 | 17 |
| *Turnix varius* | 91.9 | 3.7 | 35 | 10 | 5 | 15 | 20 |
| *Zoothera lunulata* | 115.3 | 2.1 | 28 | 10 | 9 | 21 | 8 |
| *Zosterops lateralis* | 11.6 | 2.6 | 49 | 20 | 15 | 27 | 24 |

1. Average of male and female values weighted by sample size.
2. Weighted average of reported values.
3. Frequency of occurrence of a species in different habitat types (see main text for further details).
4. Frequency of occurrence of different foraging behaviours that a species engages in
5. Frequency of occurrence of use of different foraging locations
6. Frequency of occurrence of use of different foraging substrates
7. Frequency of occurrence of different food types in the diet
