# Supplementary material for: Changes in Bird Functional Diversity across Multiple Land Uses: Interpretations of Functional Redundancy Depend on Functional Group Identity
Source: PLoS One. 2013 May 17;8(5):e63671. doi: 10.1371/journal.pone.0063671 (PMC3656964; doi:10.1371/journal.pone.0063671)
Supplement: Table S3 — Land uses for which values of each FD metric were in either the highest or lowest quartile of values for response or effect traits. (DOC) [file pone.0063671.s011.doc]

Table S3. Land uses for which values of each FD metric were in either the highest (H) or lowest (L) quartile of values for response or effect traits.

|  | Response traits | | | | Effect traits | | | |
| --- | --- | --- | --- | --- | --- | --- | --- | --- |
| Land use | FDw | FEve | FDiv | FDis | FDw | FEve | FDiv | FDis |
| VGF |  |  |  | L | H |  | L | L |
| HRF |  |  |  | L |  |  | L | L |
| HDF |  |  |  | L |  |  | L | L |
| SDF |  | H |  | L | H |  |  |  |
| BIF |  |  |  | L |  |  |  | L |
| RGW |  |  |  |  |  |  |  | L |
| BBW |  | H | H |  |  |  |  |  |
| MW |  | H |  |  |  |  |  | L |
| Mix_plan |  |  |  | L |  |  |  | L |
| RG_plan |  | H | H |  |  |  |  | L |
| BG_plan |  |  |  |  |  |  |  | L |
| Reg_mix |  | H |  |  |  |  |  | L |
| Reg_tree | L |  |  | L |  |  | L | L |
| Reg_shb |  |  |  |  |  |  | L | L |
| Urb_low |  |  |  |  |  | H |  |  |
| Pine_T2 |  |  |  | L |  |  |  | L |
| Pine_T1 |  | L |  | L | H |  | L | L |
| Pine_y |  |  |  |  |  |  |  | L |
| Pine_o |  |  |  | L |  | H |  | L |
| Urb_mid |  | H |  |  |  |  | H |  |
| Appl |  |  |  |  |  |  |  |  |
| Alm |  |  | H |  |  |  |  |  |
| Urb_high |  | H |  |  |  |  | H |  |
| Vine |  |  |  | L |  |  | H |  |

Land uses are ranked in order from lowest intensity (VGF) to highest intensity (Vine). See Table S1 for land-use codes.
